# Systematic analysis of integrated bioinformatics to identify upregulated THBS2 expression in colorectal cancer cells inhibiting tumour immunity through the HIF1A/Lactic Acid/GPR132 pathway

**DOI:** 10.1186/s12935-023-03103-5

**Published:** 2023-10-27

**Authors:** Ye Liu, Chunhui Jiang, Chunjie Xu, Lei Gu

**Affiliations:** Department of Gastrointestinal Surgery, School of Medicine, Renji Hospital, Shanghai Jiao Tong University, Shanghai, China

**Keywords:** Colorectal cancer, THBS2, Lactic acid, M2 polarisation, GPR132

## Abstract

**Background:**

THBS2, a member of the extracellular matrix glycoprotein family, can effectively inhibit tumour growth and angiogenesis. This study aimed to investigate the biological role of THBS2 in various types of cancers and the mechanisms underlying the malignant progression of colorectal cancer (CRC).

**Methods:**

THBS2 expression in pan-cancer tissues and cell lines was assessed using the HPA, TISCH and CCLE databases. The CIBERSORT, ESTIMATE, TIMER, xCell and ssGSEA (implemented using the IOBR R package) algorithms were used to calculate the proportion of tumour-infiltrating immune cells based on the expression profile of THBS2 in TCGA-COAD cohort. The clusterprofiler R package was used to implement GO and KEGG pathway enrichm SNVs were compared between the high- and low-THBS2-expression groups using the maftools R package. Additionally, immunotherapy responses were compared between the high- and low-THBS2-expression groups based on immunophenoscores (IPSs). CT26 cells were engineered to overexpress THBS2 (CT26-THBS2) to investigate its regulatory effects on HIF1 and cellular metabolism. The conditioned medium from CT26-THBS2 cells was collected to examine its effect on the M2 polarisation of RAW264.7 macrophages. Subsequently, in vitro experiments were performed to validate the inhibitory effects of M2-polarised macrophages on T-cell proliferation and cytotoxicity. A CT26-THBS2 tumour-bearing mouse model was constructed to validate the impact of high THBS2 expression in tumour cells on the tumour microenvironment in vivo.

**Results:**

THBS2 expression was upregulated in a majority of tumours, including COAD, and was positively associated with ESTIMATEScore, ImmuneScore and StromalScore. Furthermore, THBS2 expression was positively associated with angiogenesis and epithelial–mesenchymal transition and negatively associated with DNA repair, cell cycle and DNA replication in most tumours. THBS2 expression was considerably associated with progression-free interval (PFI) and positively associated with MSI in COAD. THBS2 methylation levels were remarkably lower in COAD tissues than in healthy tissues. The high expression of THBS2 in CT26 cells remarkably promoted the nuclear translocation of HIF1 and consequently enhanced lactate metabolism in cells. In vitro and in vivo experiments revealed that lactate released by tumour cells promoted M2 polarisation of macrophages, leading to inhibition of T-cell proliferation and cytotoxicity.

**Conclusions:**

THBS2 expression is associated with PFI, immune cell infiltration, immune regulation, cell death, cell migration, epithelial–mesenchymal transition, angiogenesis and genomic variations in COAD. THBS2 may serve as a biomarker for immunotherapy in COAD. Upregulated THBS2 expression in CRC cells inhibits anti-tumour immunity through the HIF1A/lactic acid/GPR132 pathway.

**Supplementary Information:**

The online version contains supplementary material available at 10.1186/s12935-023-03103-5.

## Introduction

Colorectal cancer (CRC) is a prevalent malignancy arising from abnormal cells in the colonic mucosa [1, 2]. Genetic mutations and aberrant cell proliferation contribute to the progression of benign adenomas to adenomatous polyps, eventually leading to malignancy. CRC manifests with symptoms such as irregular bowel movements, abdominal pain, bloating, altered bowel habits, rectal bleeding, anaemia, weight loss and fatigue. Because early-stage CRC is often asymptomatic, early diagnosis of CRC is necessary for improving the prognosis [3, 4].

The platelet reactive protein (THBS) family consists of five members including THBS1, THBS2, THBS3, THBS4 and THBS5, which are calcium-associated glycoproteins with multiple structural domains. Based on the oligomerisation status and different structural domains, these proteins can be classified as trimers and pentamers. In particular, THBS1 and THBS2 are trimers, whereas the other three proteins are pentamers [5].

THBS2 is widely expressed across various tissues and cell types and participates in crucial biological processes, including extracellular matrix formation, cell proliferation, migration, apoptosis and angiogenesis [6, 7]. Studies investigating the expression and role of the THBS2 gene in different tumour types have revealed controversial results. Some studies have shown that THBS2 is upregulated in non-small cell lung cancer [8], breast cancer [9] and CRC [6] but downregulated in cervical cancer [10]. Although THBS2 has been reported to be involved in some specific tumours, no studies have reported a systemic pan-cancer analysis of THBS2 to date. High THBS2 expression has been associated with low survival rates and disease progression in CRC, indicating the potential of THBS2 as a prognostic biomarker. THBS2 expression independently reflects prognosis in patients with CRC. In particular, patients with high THBS2 expression have considerably lower survival rates than those with low THBS2 expression [11–13]. However, further investigation is warranted to examine the clinical significance of THBS2 in CRC and understand the mechanisms through which THBS2 influences the prognosis of CRC.

Hypoxia-inducible factor 1 (HIF1) is a transcription factor that is crucial for cellular responses to hypoxia [14, 15]. It consists of two subunits, HIF1α and HIF1β. HIF1β is a conventional nuclear transcription factor, whereas HIF1α is an oxygen concentration-related subunit. Under normoxic conditions, HIF1α is regulated through an oxygen-dependent protein degradation mechanism, resulting in its low levels. However, under hypoxic conditions (such as hypoxia), the degradation of HIF1α is inhibited, resulting in its stabilisation and nuclear translocation [16, 17]. In the nucleus, HIF1α interacts with HIF1β to form the HIF1 complex, which promotes the transcription of downstream genes. HIF1 is critically involved in various physiological and pathological processes, including embryonic development, tumour progression, cardiovascular disease development and inflammation [18, 19]. We have previously demonstrated that high expression of THBS2 is strongly associated with cellular anaerobic metabolism [20]. In this study, we compared the expression of THBS2 across various cancer types (pan-cancer analysis) and examined its relationship with the immune microenvironment, genomic mutations, methylation status and prognosis. In addition, we investigated the mechanisms through which THBS2 contributes to the malignant progression of CRC by regulating the immune microenvironment. Altogether, the findings of this study provide valuable insights into the relationship between THBS2 and HIF-1 and introduce a novel biomarker for targeted therapy in CRC.

## Methods and materials

### Extraction and pre-processing of gene expression data

The gene expression profiles of various cancer types were retrieved from TCGA database. Additionally, clinical information was obtained using the TCGAbiolinks R package. FPKM values were converted to TPM values, and log_2_(TPM + 1) was used for subsequent analysis. Based on the corrected survival information from TCGA database [21], samples with an overall survival (OS) of < 30 days were excluded to ensure reliability. Mutation data were extracted from the COAD cohort and used for genomic variation analysis. The clinical information of patients in TCGA-COAD cohort is shown in Table 1.

**Table 1:** TCGA-COAD cohort sample clinical information

TCGA cohort	Group information	Number of samples
OS	0	329
	1	95
Age	Age≥60	305
	Age < 60	119
Gender	Female	195
	Male	229
Stage	Stage I/II	232
	Stage III/IV	181
DSS	0	350
	1	58
DFI	0	162
	1	23
PFI	0	309
	1	115

The RNA-sequencing data of cell lines were extracted from the CCLE database (https://sitesbroadinstitute.org/ccle). The methylation data of THBS2 in pan-cancer were extracted from TCGA datasets in the eBioPortal database (https://www.cbioportal.org/). The RNA-sequencing data of the HPA and GTEx cohorts were obtained from the HPA database (https://www.proteinatlas.org/). Additionally, the immunophenoscores (IPSs) of 20 TCGA cohorts were obtained from TCIA database (https://imaging.cancer.gov/).

The uroepithelial carcinoma dataset Imvigor210 [22] was downloaded and processed as the PD-L1 immunotherapy cohort. The gene expression and clinical data of samples in the Imvigor210 dataset were normalised to raw counts using the DEsea2 R package, and the data were subsequently converted to TPM values. The expression profiles and clinical information of samples in the GSE91061 and PMC6898788 datasets were extracted from the GEO database [23] as the CTLA4 and PD1 immunotherapy cohorts, respectively. The information of samples in the three immunotherapy datasets is provided in Table 2.

**Table 2:** Immunotherapy cohort sample information

Cohort	Group	Group information	Number of samples
IMvigor210	Response	CR/PR	68
		SD/PD	230
PMC6898788	Response	CR/PR	47
		SD/PD	72
GSE91061	Response	CR/PR	10
		SD/PD	39

### Gene set acquisition

The HALLMARK and KEGG gene sets were obtained from MsigDB. A list of ferroptosis-related genes was obtained from FerrDB (http://www.zhounan.org/ferrdb/current/). A list of immune regulators was obtained from TISIDB. Additionally, an immune function gene set [24] and a list of cuproptosis-related genes [25] were derived from published literature.

### Differential expression analysis

The limma [26] R package was used to screen for differentially expressed genes between the high- and low-THBS2-expression groups in TCGA-COAD cohort. The screening criteria were set as adjusted *p-*values of < 0.05 and|log_2_FC| values of > 0.5.

### Single-gene survival analysis

Based on the median expression of the THBS2 gene, tumour samples in each TCGA cohort were divided into high- and low-THBS2-expression groups. Kaplan–Meier curves were plotted to compare survival between the two groups. Significant differences were evaluated using the log-rank test, with *p*-values of < 0.05 indicating statistical significance.

### Estimation of the proportion of tumour-infiltrating immune cells and immune scores

Five algorithms including CIBERSORT [27], ESTIMATE, TIMER, xCell and ssGSEA (implemented in the IOBR R package) were used to calculate the proportion of tumour-infiltrating immune cells based on the expression profiles of samples in TCGA-COAD cohort. For pan-cancer analysis, the ESTIMATE, xCell and ssGSEA algorithms were used to assess the correlation between THBS2 expression and immune cell infiltration in various TCGA cohorts. Based on the marker genes of 28 immune cell types, the enrichment scores (degree of infiltration) of the 28 cell types in each sample were assessed using the ssGSEA algorithm in the GSVA R package.

### Gene set enrichment analysis

The ssGSEA algorithm was implemented in the GSVA R package to evaluate the enrichment scores of samples in TCGA cohorts based on the gene expression data. GO and KEGG pathway enrichment analyses were performed using the clusterprofiler R package, with *p*-values of < 0.05 indicating significantly enriched gene sets. The top 10 gene sets with significant enrichment (*p* < 0.05) were visualised on bubble plots.

### Genomic SNV analysis

Based on the MAF file of somatic mutations in TCGA-COAD cohort, a covariance heat map demonstrating the top 10 mutated genes in different subgroups was plotted using the ‘somaticInteractions()’ function in the maftools R package. Additionally, SNVs were compared among groups, and the results were visualised on a waterfall plot generated using the ‘oncoplot()’ function. Finally, the MAF data of the high- and low-THBS2-expression groups were analysed using the ‘mafCompare()’ function to identify genes with significant differences in mutations between the two groups.

### Prediction of immunotherapy response

IPSs were used to examine the immunogenicity of a tumour and predict its response to immunotherapy. Differences in IPSs between the high- and low-THBS2-expression groups were statistically estimated to compare immunotherapy responses between the two groups.

### Cell culture and transfection

The murine cell lines CT26 and RAW264.7 were previously maintained in our laboratory. The cells were cultured in Dulbecco’s modified Eagle medium (DMEM) supplemented with 10% foetal bovine serum (FBS) and maintained in a humidified incubator with 5% CO_2_ at a temperature of 37 °C. The cells were passaged after they reached 80% confluence. A plasmid encoding mouse THBS2 gene, an shRNA targeting mouse HIF1 (sh-HIF1) and their respective controls were synthesised by Genechem (Shanghai, China). Cells were transfected with the shRNA, plasmid or their respective controls using the Lipofectamine 3000 reagent (Thermo Fisher Scientific). After 24 h, transfection efficiency was assessed via western blotting. Lipofectamine 3000 was used to co-transfect 293 T cells with plasmids, including lentiviral vectors, to generate lentiviruses. After 48 h, the supernatant containing the viruses was collected for subsequent infection. For transduction, CT26 cells were incubated with the lentiviruses overnight in a humidified incubator at 37 °C. Thereafter, transduced cells were selected in the presence of 1.5-μg/mL puromycin for 24 h.

### Establishment of tumour-bearing mouse models and assessment of tumour size

Female Balb/c mice (age, 6 weeks) were purchased from Life River Laboratory (Beijing, China). All experiments involving animals were performed following the laboratory guidelines for animal care and use, adhering to the ARRIVE guidelines. The mice were housed indoors under standard conditions, with a 12/12-h light/dark cycle. Tumour size was assessed every 2 days after CT26 or CT26-THBS2 cells were inoculated into the mice. A calliper was used to measure the tumour area (length and width), which was expressed as mm^2^. Each animal experiment was repeated two or three times, with a minimum of three mice per group in each replicate. After the animals were euthanised, tumours smaller than 150 mm^2^ were excised under specific conditions and immediately frozen at −80 °C for further analysis.

### Immunofluorescence analysis

CT26 cells cultured on coverslips were rinsed with PBS, fixed with PFA and blocked with 5% BSA. Subsequently, the cells were incubated with anti-HIF1 antibody overnight at 4 °C. The following day, the cells were incubated with appropriate secondary antibodies at room temperature. After the cells were washed with PBS, the nuclei were stained with Hoechst for 5 min. Finally, the coverslips were mounted, and images were captured using a confocal microscope.

### qRT-PCR

Total RNA was extracted from CT26 cells using TRIzol reagent (Invitrogen). The extracted RNA was reverse transcribed to cDNA using either the TaqMan™ Advanced miRNA cDNA Synthesis Kit or the First Strand cDNA Synthesis Kit. Thereafter, qPCR was performed on the ABI 7500 Fast Real-Time PCR System using the SYBR Green OCR Kit. To ensure accurate comparisons, all data were normalised to the expression of U6, which served as the internal control.

### Extracellular flux analysis

The Seahorse XF24 Extracellular Flux Analyzer was used to assess glycolysis and mitochondrial respiration as described in a previous study [28]. In particular, Real-time alterations in the extracellular acidification rate (ECAR) and oxygen consumption rate (OCR) of cells were continuously monitored.

### Lactate measurement

Cells were cultured in an equivalent volume of medium for 3 days. Subsequently, the cells were harvested and centrifuged at 10,000 × g for 10 min in a 5-mL centrifuge tube. The supernatant was collected for subsequent analysis. Lactate levels in the supernatant were measured using the Amplite Fluorimetric l-Lactate Assay Kit according to the manufacturer’s instructions. The relative fluorescence intensity was determined using a multifunctional microplate reader.

### Western blotting

Total proteins were extracted from CT26 cells using RIPA buffer. The extracted proteins were separated on a 10% sodium dodecyl sulphate–polyacrylamide gel and transferred onto a PVDF membrane. To prevent non-specific binding, the membrane was blocked with 5% milk at room temperature for 1 h. Subsequently, it was incubated with primary antibodies against THBS2 (1:1,000; ab112543; Abcam, Cambridge, UK), HIF1 (1:500; ab51608; Abcam, Cambridge, UK), lamin B (1:500; ab32535; Abcam, Cambridge, UK) and GAPDH (1:1000; ab8245; Abcam, Cambridge, UK) overnight at 4 °C. The following day, the membrane was washed thrice with TBST and incubated with secondary antibodies (1:2000) for 1 h at room temperature. After the membrane was washed thrice with TBST, protein bands were visualised using an enhanced chemiluminescence reagent. The band intensities were measured using the UN-SCAN-IT gel analysis software, and the expression of target proteins was normalised to that of GAPDH.

### Flow cytometry

Cultured RAW264.7 cells were centrifuged and resuspended in an assay buffer. Before flow cytometric analysis, the cells were washed twice with the assay buffer. The differentiation status of the cells was assessed using anti-CD163 and anti-CD206 antibodies.

CD8 + T cells were isolated from the peripheral blood of OT-1 mice using sorting techniques and added to a co-culture system. After the co-culture, the cells were collected from Transwell inserts. Cell proliferation was assessed using the CFSE assay kit, whereas cell apoptosis was assessed using the 7-AAD assay kit. The release of interferons (IFNs) from T cells was evaluated using anti-IFN antibodies.

For flow cytometric analysis of mouse tumour tissues, cells were dissociated using trypsin and resuspended in an assay buffer at a concentration of 1 × 10^6^ cells/mL. Before flow cytometric analysis, the cells were washed twice with the assay buffer. Cell apoptosis was assessed using the 7-AAD assay kit. To identify specific cell populations, antibodies against CD163, CD206, CD3, CD4, CD8, F4/80, CD11b, TIM3, Foxp3 and PD1 were used to label immune cells. Flow cytometric data were analysed using the FlowJo software.

### Statistical analysis

Statistical analysis was performed using the GraphPad Prism software (GraphPad 8). Data with a normal distribution were expressed as the mean ± SEM. In the case of a single variable, differences among three or more groups were evaluated using one-way analysis of variance (ANOVA) followed by Tukey’s post-hoc test. In the case of two variables, differences among three or more groups were evaluated using two-way ANOVA with Tukey’s post-hoc test. The value of n represented the number of independent experimental replicates for each graph. Statistical significance was denoted as follows: *p < 0.05; **p < 0.01; ***p < 0.001. Non-significant differences were denoted as ‘ns’ in the graphs.

## Results

### Biological characteristics of THBS2 in pan-cancer

#### Expression of THBS2 in pan-cancer

**Fig. 1:**
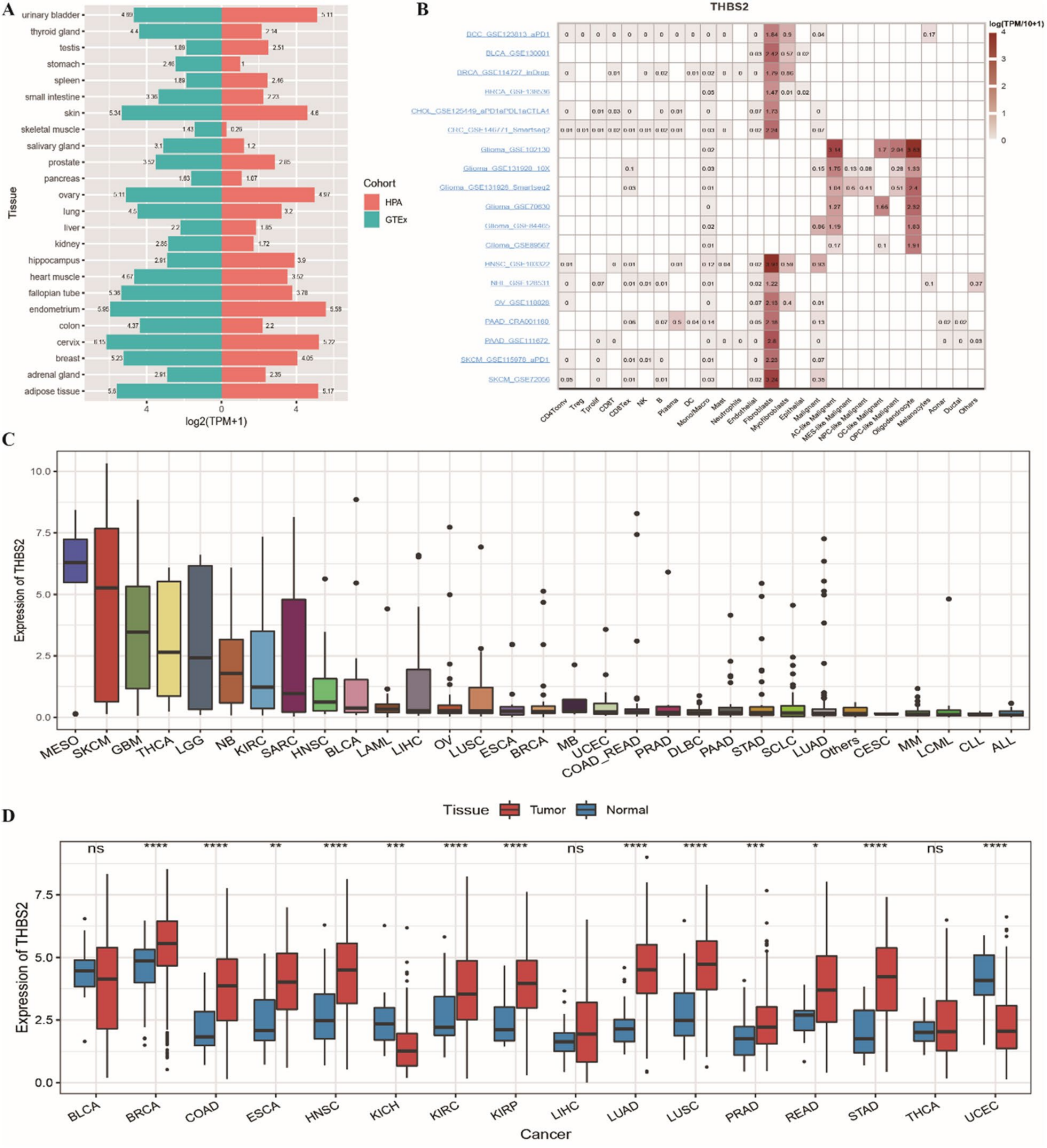
Expression of THBS2 in pan-cancer. **A** Histogram of THBS2 expression in the HPA and GTEx cohorts. Horizontal coordinates represent TPM values; the right side of 0 represents the HPA cohort; the left side represents the GTEx cohort and vertical coordinates represent tissue types; **B** Heat map demonstrating THBS2 expression in each cell type in pan-cancer single-cell datasets; **C** Box plot demonstrating THBS2 expression in different cancer cell lines in the CCLE database; D: Box plots demonstrating dysregulation of the THBS2 gene in 16 cancer types; red represents tumour tissues; blue represents normal tissues and * represents significant differences

The expression of THBS2 in 22 tissue samples in the HPA and GTEx cohorts was examined using the HPA database. The results indicated that THBS2 was significantly upregulated in various tissues, including cervical, endometrial and skin tissues (Fig. 1A). Furthermore, the expression of THBS2 was examined in various cell types using the TISCH database. Among the cells exhibiting more significant THBS2 expression (log[TPM/10 + 1] > 1), fibroblasts had remarkably high expression of THBS2 in most cancers (Fig. 1B). Gene expression data extracted from the CCLE database indicated that the expression of THBS2 was higher in MESO, SKCM, GBM, THCA and LGG cells (Fig. 1C). A box plot was generated to demonstrate the differential expression of THBS2 between tumour and normal tissues in 16 solid tumour cohorts. In particular, THBS2 was considerably downregulated in KICH and UCEC and upregulated in other tumour types including COAD (Fig. 1D).

#### THBS2 is associated with the immune microenvironment in pan-cancer

**Fig. 2:**
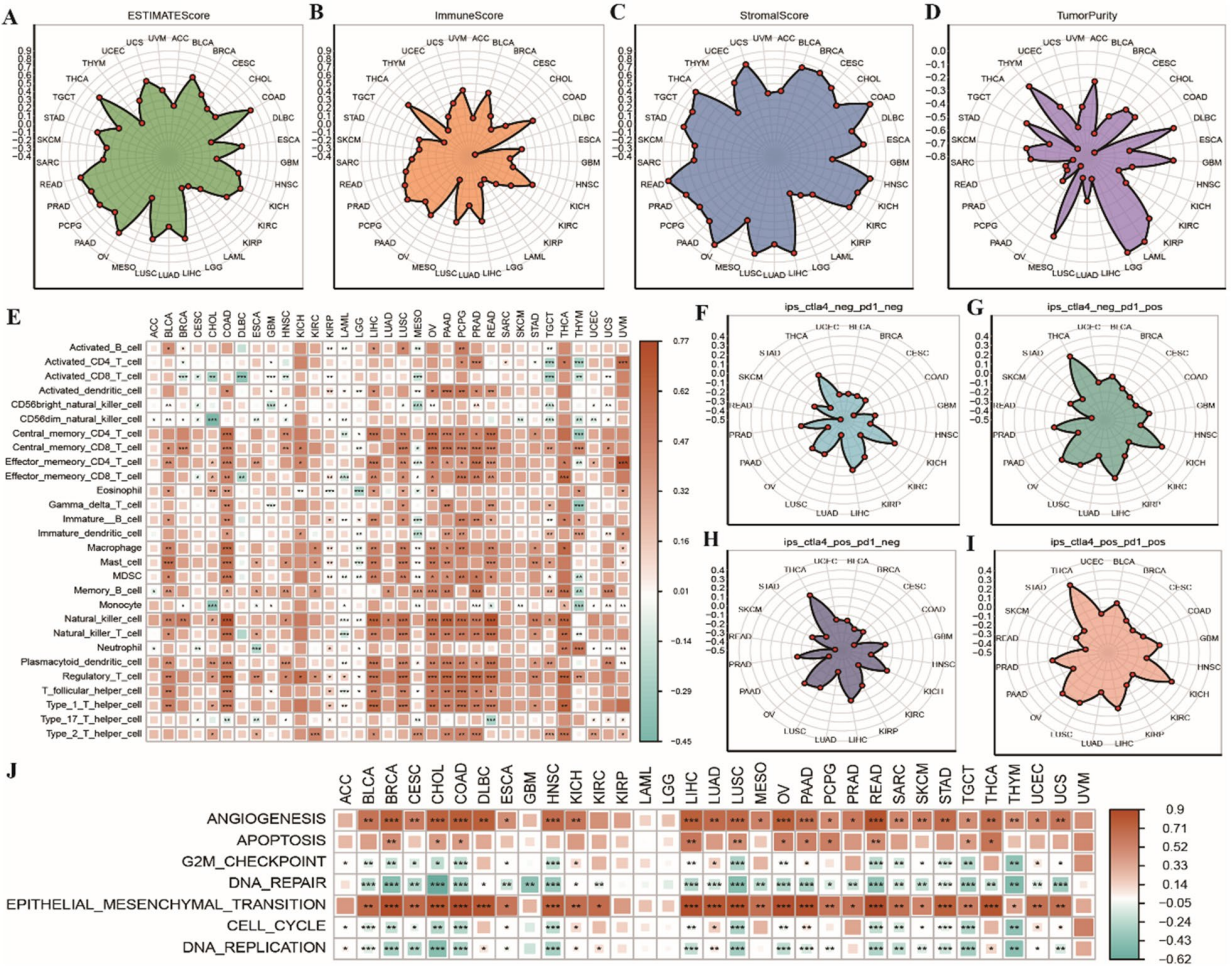
Correlation between THBS2 expression and the immune microenvironment in pan-cancer. **A**–**D** Radar plot demonstrating the correlation between THBS2 expression and four types of immune-related scores in pan-cancer, with increasing correlation coefficients shown in ascending order; **E** Heat map demonstrating the correlation between THBS2 expression and the proportion of 28 types of tumour-infiltrating immune cells in pan-cancer, with the colour of the dots indicating a high correlation and positive or negative correlation and * indicating significance; **F**–**I** Radar plot demonstrating the correlation between IPSs and THBS2 expression in pan-cancer; **J** Heat map demonstrating the correlation between multiple cell functions and THBS2 expression in pan-cancer

Four types of immune-related scores were calculated using the ESTIMATE algorithm. Subsequently, the relationship between THBS2 expression and these immune-related scores was assessed. The results revealed that THBS2 expression was positively correlated with ESTIMATEScore, ImmuneScore and StromalScore. THBS2 expression was strongly correlated with tumour purity in THCA, COAD, READ, LICH and KICH, whereas it was negatively correlated with tumour purity in THCA, DLBC, LGG and GBM (Fig. 2A–D). A heat map was generated to demonstrate the correlation between THBS2 expression and the proportion of 28 types of tumour-infiltrating immune cells. In particular, THBS2 expression was positively associated with the proportion of memory T cells, NK cells and regulatory T cells in pan-cancer and negatively associated with the proportion of activated CD8 T cells in CHOL, DLBC, MESO and TGCT (Fig. 2E). Radar plots was generated to demonstrate the correlation between IPSs and THBS2 expression in pan-cancer. The plot showed that THBS2 expression was positively associated with IPSs in THCA, KICH and LIHC and negatively associated with IPSs in COAD, READ and SKCM (Fig. 2F–I). In addition, THBS2 expression was positively associated with angiogenesis and epithelial–mesenchymal transition and negatively associated with DNA repair, cell cycle and DNA replication in most cancer types (Fig. 2J).

#### THBS2 is associated with prognosis in pan-cancer

**Fig. 3:**
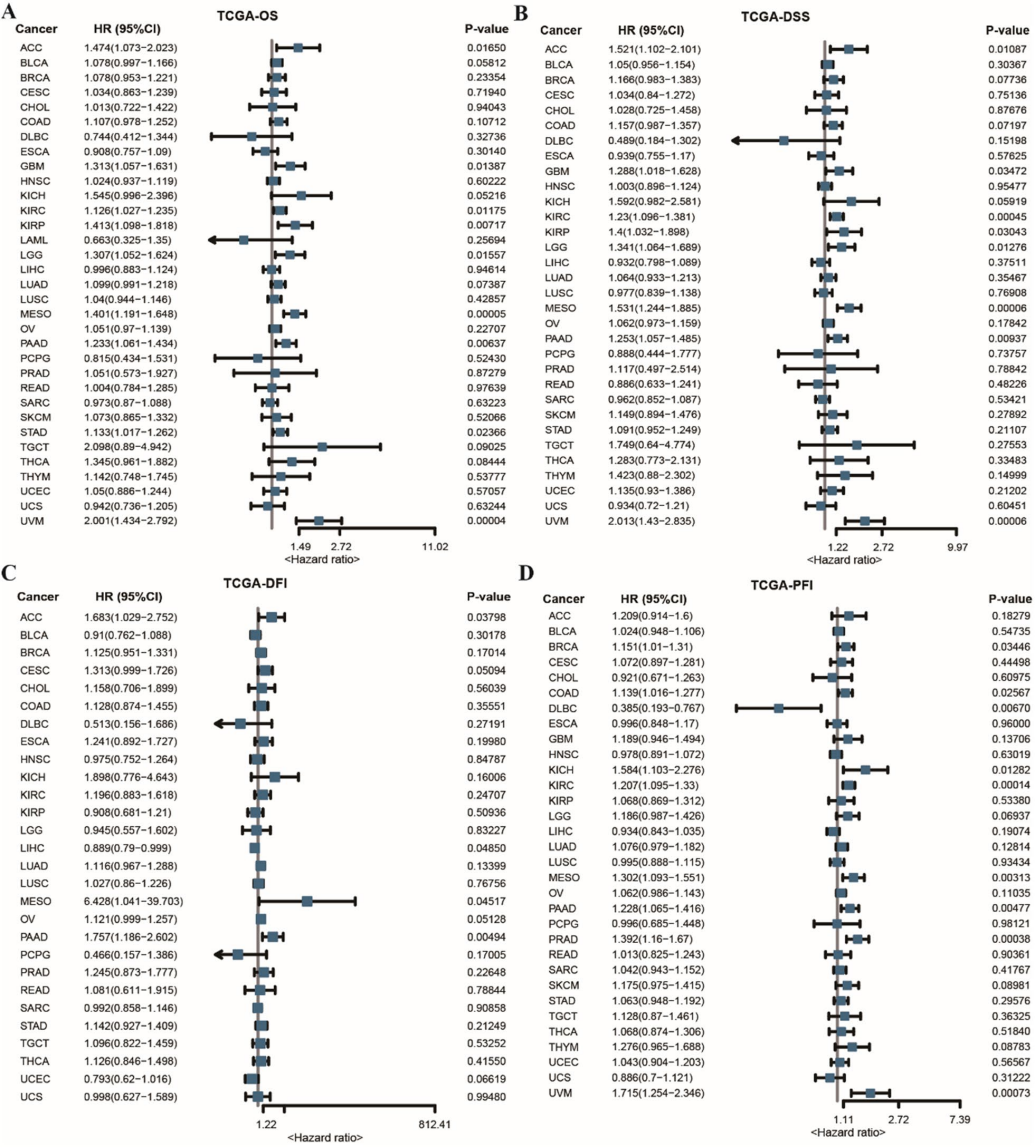
THBS2 expression is associated with prognosis in pan-cancer. **A** Forest plot demonstrating the relationship between THBS2 expression and OS as assessed via univariate Cox analysis; **B** Forest plot demonstrating the relationship between THBS2 expression and DSS as assessed via univariate Cox analysis; **C** Forest plot demonstrating the relationship between THBS2 expression and DFI as assessed via univariate Cox analysis; **D** Forest plot demonstrating the relationship between THBS2 expression and PFI as assessed via univariate Cox analysis

Univariate Cox analysis was performed to examine the relationship between THBS2 expression and prognosis in pan-cancer based on OS, DSS, DFI and PFI data. DFI data were not available in the LAML, SKCM, UVM, GBM and THYM cohorts, whereas DSS and PFI data were not available in the LAML cohort. Forest plots were generated to visualise the results of univariate Cox analysis. In particular, THBS2 expression was remarkably associated with OS in UVM, LGG, GBM, PAAD, KIRP, MESO, ACC, STAD and KIRC (Fig. 3A) and with DSS in UVM, LGG, GBM, PAAD, KIRP, MESO, ACC, STAD and KIRC (Fig. 3B). Furthermore, THBS2 expression was significantly associated with DFI in ACC, LIHC, MESO and PAAD (Fig. 3C) and with PFI in PRAD, UVM, DLBC, BRCA, KICH, MESO, COAD, KIRC and PAAD (Fig. 3D).

#### Genomic instability of THBS2 in pan-cancer

**Fig. 4:**
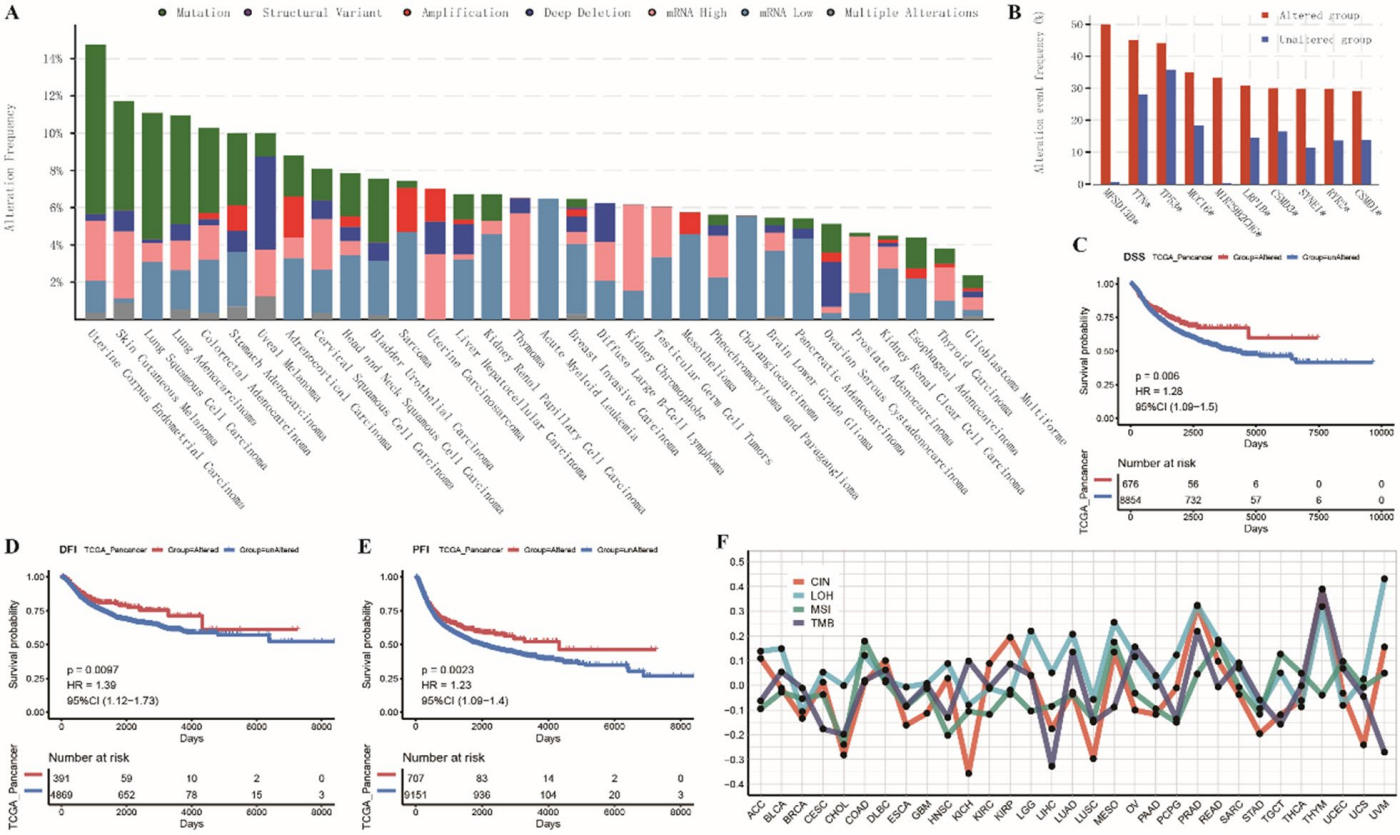
Genomic instability of THBS2 in pan-cancer. **A** Histogram demonstrating the distribution of mutation types of THBS2 in pan-cancer; **B** Gene mutations significantly associated with THBS2 mutations in pan-cancer; **C**–**E** KM curves demonstrating the relationship between THBS2 mutations and survival based on DSS, DFI and PFI data in pan-cancer; **F** Histogram demonstrating the correlation between THBS2 expression and CIN, LOH, MSI or TMB in pan-cancer

Mutations in genetic loci can lead to transcriptional errors and genomic instability, affecting tumorigenesis. The cBioPortal database was used to analyse THBS2 mutations and evaluate the frequency of various mutation types in pan-cancer. The results revealed that THBS2 had a higher frequency of mutation in UCEC, SKCM, LUSC, LUAD and COAD and a higher frequency of copy number amplification in STAD, ACC, SARC, UCS and MESO (Fig. 4A). High-frequency mutations significantly associated with THBS2 mutations in pan-cancer are shown in Fig. 4B. The mutation frequency of the MFSD138 gene was remarkably higher in the THBS2-mutation group and lower in the non-THBS2-mutation group. Based on OS, DSS, DFI and PFI data, survival was compared between the THBS2-mutation and non-THBS2-mutation groups in pan-cancer. KM curves indicated that survival was significantly better in the THBS2-mutation group (Fig. 4C–E). However, OS was not significantly different between the two groups. Furthermore, the correlation of THBS2 expression with CIN, LOH, MSI and TMB was assessed in pan-cancer. The results revealed a positive correlation between MSI and THBS2 expression in COAD and a negative correlation between CIN and THBS2 expression in KIHC and LUSC. In addition, THBS2 expression was positively correlated with LOH in UVM, PRAD and THYM. TMB was negatively associated with THBS2 expression in LIHC and UVM (Fig. 4F).

#### THBS2 methylation in pan-cancer

**Fig. 5:**
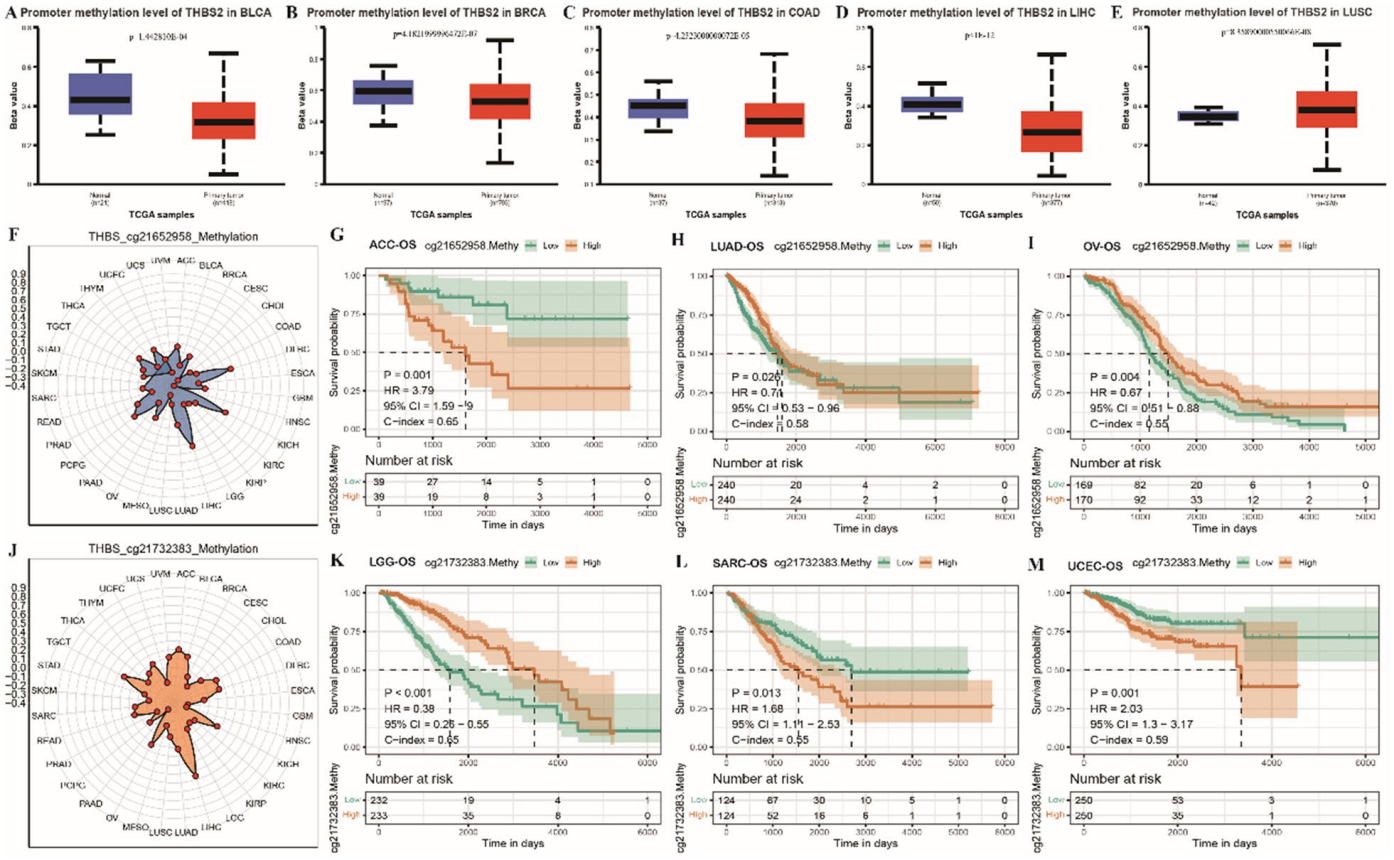
THBS2 methylation in pan-cancer. **A**–**E** Box plots demonstrating differences in THBS2 methylation levels between normal and tumour tissues in pan-cancer; red represents tumour tissues; blue represents normal tissues; p-values represent significant differences; **F** Radar plot demonstrating the correlation between methylation levels and THBS2 expression at cg21652958 locus in pan-cancer; **G**–**I** KM curves demonstrating OS in the high- and low-cg21652958-methylation groups; orange represents the high-cg21652958-methylation group; green represents the low-cg21652958-methylation group; p-values represent significant differences; **J** Radar plot demonstrating the correlation between the methylation levels of cg21732383 and THBS2 expression in pan-cancer; **K**–**M** Radar plot demonstrating differences in OS between the high- and low-cg21732383-methylation groups based on KM curves

Methylation has been associated with the development, mutagenesis and immune tolerance of various cancers. The methylation levels of THBS2 were compared between tumour and normal tissues using the UALCAN database (https://ualcan.path.uab.edu/). The results showed that THBS2 methylation levels were remarkably lower in BLCA, BRCA, COAD, LIHC and LUSC tissues than in normal tissues (Fig. 5A–E). Further analysis of the correlation between the cg21652958 methylation site in the 5'-UTR of THBS2 and THBS2 expression showed that the methylation levels of cg21652958 were significantly positively correlated with THBS2 expression in DLBC, KICH and LIHC (Fig. 5F). KM curves were generated to compare OS between the low- and high-cg21652958-methylation groups in ACC, LUAD and OV. The results revealed that OS was better in the low-cg21652958-methylation group in ACC and in the high-cg21652958-methylation group in LUAD and OV (Fig. 5G–I). Further analysis of the relationship between THBS2 expression and the cg21732383 methylation site in TSS1500 (1500-bp upstream of the transcription initiation site) showed that the methylation levels of cg21732383 were most strongly correlated with THBS2 expression in LIHC (Fig. 5J). KM curves showed that OS was better in the high-cg21732383-methylation group in LGG and in the low-cg21732383-methylation group in SARC and UCEC (Fig. 5K–M).

### Prognostic characterisation and role of THBS2 in COAD

#### Expression of THBS2 in COAD

The abovementioned results suggest that the expression, genomic variations and methylation of THBS2 are closely related to tumour prognosis.

**Fig. 6:**
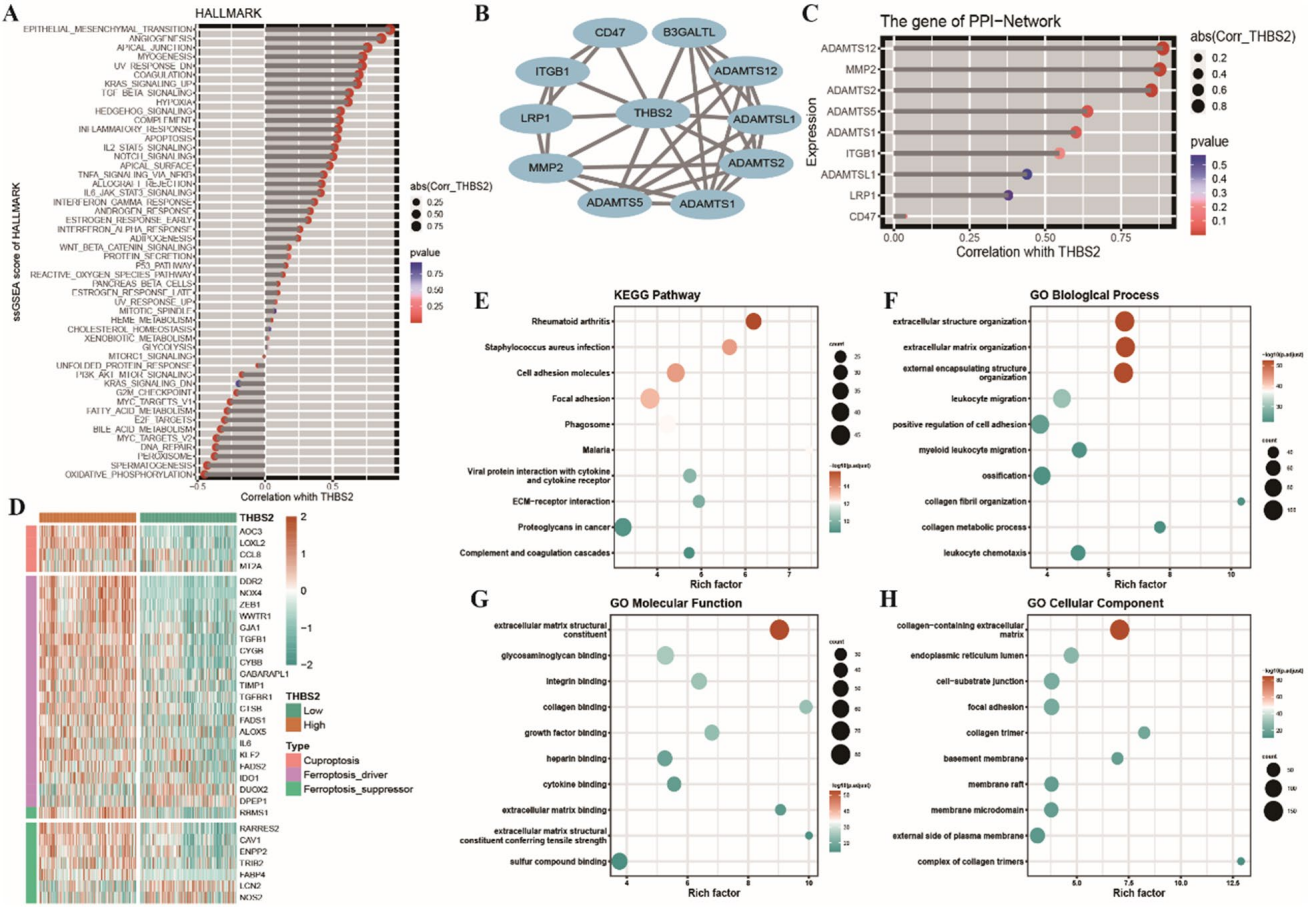
Expression of THBS2 in COAD. **A** Stick plot demonstrating the correlation between THBS2 expression and the enrichment scores of the HALLMARK gene set; the size of the dots represents a strong correlation; the right side represents a positive correlation; the left side represents a negative correlation; the colour represents p-values; the smaller the p-value, the more significant the correlation; **B** Protein–protein interaction network of THBS2 constructed using the STRING database; **C** Stick plots demonstrating the correlation between the expression of THBS2 and that of other proteins in the network; **D** Heat map of differential expression of copper death- and iron death-related genes between the high- and low-THBS2-expression groups; **E** Bubble plots of KEGG, GO-BP (biological process), GO-MF (molecular function) and GO-CC (cellular component) enrichment analyses of marker genes in the high-THBS2-expression group; the size of the dots represents the number of enriched differentially expressed genes; the colour represents the significance of the enrichment

To investigate the impact of THBS2 on colon cancer, its expression pattern was examined in TCGA-COAD cohort and its relationship with the enrichment scores of the HALLMARK gene set was assessed. The results showed that THBS2 expression was positively associated with epithelial–mesenchymal transition and angiogenesis and negatively associated with DNA repair, oxidative phosphorylation, spermatogenesis and peroxisome (Fig. 6A). The protein–protein interaction network of THBS2 in COAD is shown in Fig. 6B. Based on this network, the expression of THBS2 was positively associated with that of ADAMTS12, MMP2 and ADAMTS2 (Fig. 6C). Based on the median expression of THBS2 (3.92), TCGA-COAD cohort was divided into high- and low-THBS2-expression groups. A total of 1052 differentially expressed genes were identified between the two groups, which included 4 copper death-related genes and 28 iron death-related genes. As shown in the heat map in Fig. 6D, LCN2 and NOS2 were included in the THBS2 group, whereas the remaining genes were upregulated. Furthermore, the 992 upregulated genes in the high-THBS2-expression group were subjected to KEGG and GO functional enrichment analyses. KEGG analysis revealed that the genes were primarily enriched in pathways associated with rheumatoid arthritis, cell adhesion molecules, phagosomes, viral protein interactions with cytokines and cytokine receptors, ECM–receptor interactions and proteoglycans in cancer. GO analysis revealed that the genes were enriched in biological processes such as cell structural organisation, cell migration and leukocyte chemotaxis; cellular components such as the extracellular matrix and endoplasmic reticulum lumen and molecular functions such as growth factor binding and cytokine binding (Fig. 6E–H). In addition, 60 genes were down-regulated in the high THBS2-expression group. These down-regulated genes were further used for enrichment analyses. As shown in the figure, there are no significant enrichment results obtained in KEGG pathway analysis (Additional file 1: Fig. S1). The possible reason for this is due to the low number of genes involved in the enrichment analysis. Therefore, we were more concerned regarding the expression profiles of the upregulated genes in the high-THBS2-expression group.

#### Differences in immunological characteristics between high- and low-THBS2-expression groups

**Fig. 7:**
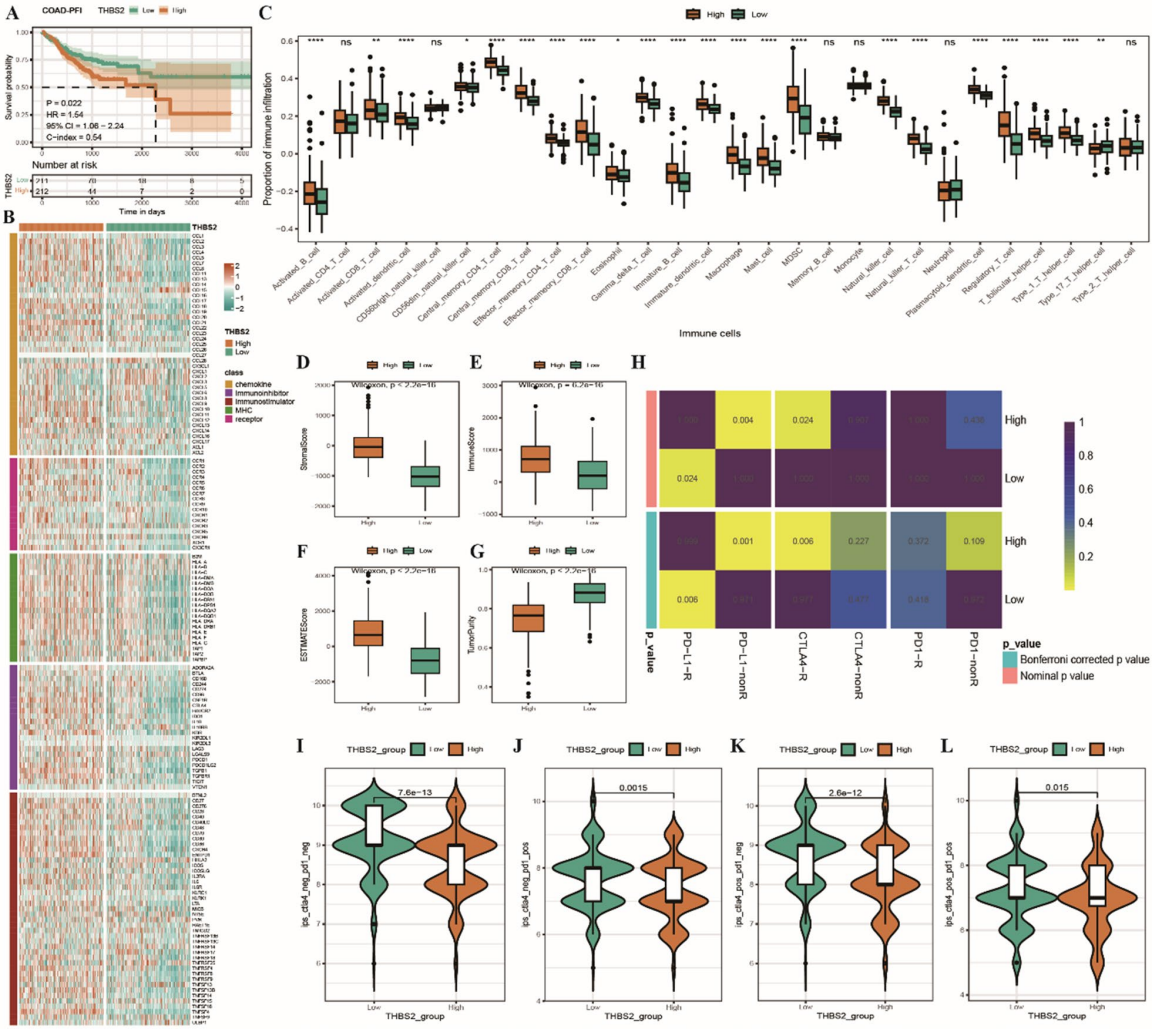
Immunological differences between high- and low-THBS2-expression groups. **A** KM curves demonstrating PFI in the high- and low-THBS2-expression groups; B: Heat map demonstrating the differential expression of immunomodulators between the high- and low-THBS2-expression groups; **C** Box plot demonstrating differences in the proportion of 28 types of tumour-infiltrating immune cells between the two THBS2 groups; **D**–**G** Box plots demonstrating differences in the four types of scores between the two THBS2 groups as evaluated using the ESTIMATE algorithm; **H** Heat map of similarity between the high- and low-THBS2-expression groups and immunotherapy response groups (with and without response subgroups) obtained from SubMap analysis; **I**, **J** Violin plots demonstrating differences in IPSs between the high- and low-THBS2-expression groups

KM curves revealed that PFI was worse in the high-THBS2-expression group, indicating that THBS2 expression was remarkably associated with prognosis in COAD (Fig. 7A). To investigate the mechanisms through which THBS2 affects the prognosis of COAD through immune-related factors, the expression of immunomodulators was examined in the two THBS2 groups. The results indicated that the expression of immunomodulators was higher and that of CCL28, CXCL1, CXCL2 and CXCL3 chemokines was lower in the high-THBS2-expression group (Fig. 7B). Furthermore, the proportion of 28 types of tumour-infiltrating immune cells was compared between the two THBS2 groups. As shown in the box plot in Fig. 7C, the degree of immune cell infiltration was lower in the low-THBS2-expression group than in the high-THBS2-expression group. The ESTIMATE algorithm was used to calculate stromal, immune and ESTIMATE scores in the two groups. The three types of scores were considerably lower in the low-THBS2-expression group than in the high-THBS2-expression group (Fig. 7D–G). Similarity *p*-values were calculated in the two THBS2 groups and three immunotherapy cohorts using SubMap. As shown in the heat map in Fig. 7H, the low-THBS2-expression group was more responsive to PD-L1 immunotherapy, whereas the high-THBS2-expression group was more responsive to CTLA4 immunotherapy. In addition, all four IPSs were considerably higher in the low-THBS2-expression group than in the high-THBS2-expression group (Fig. 7I–L). These results indicated that patients in the low-THBS2-expression group were more likely to benefit from immunotherapy.

#### THBS2 is associated with genomic mutations in COAD

**Fig. 8:**
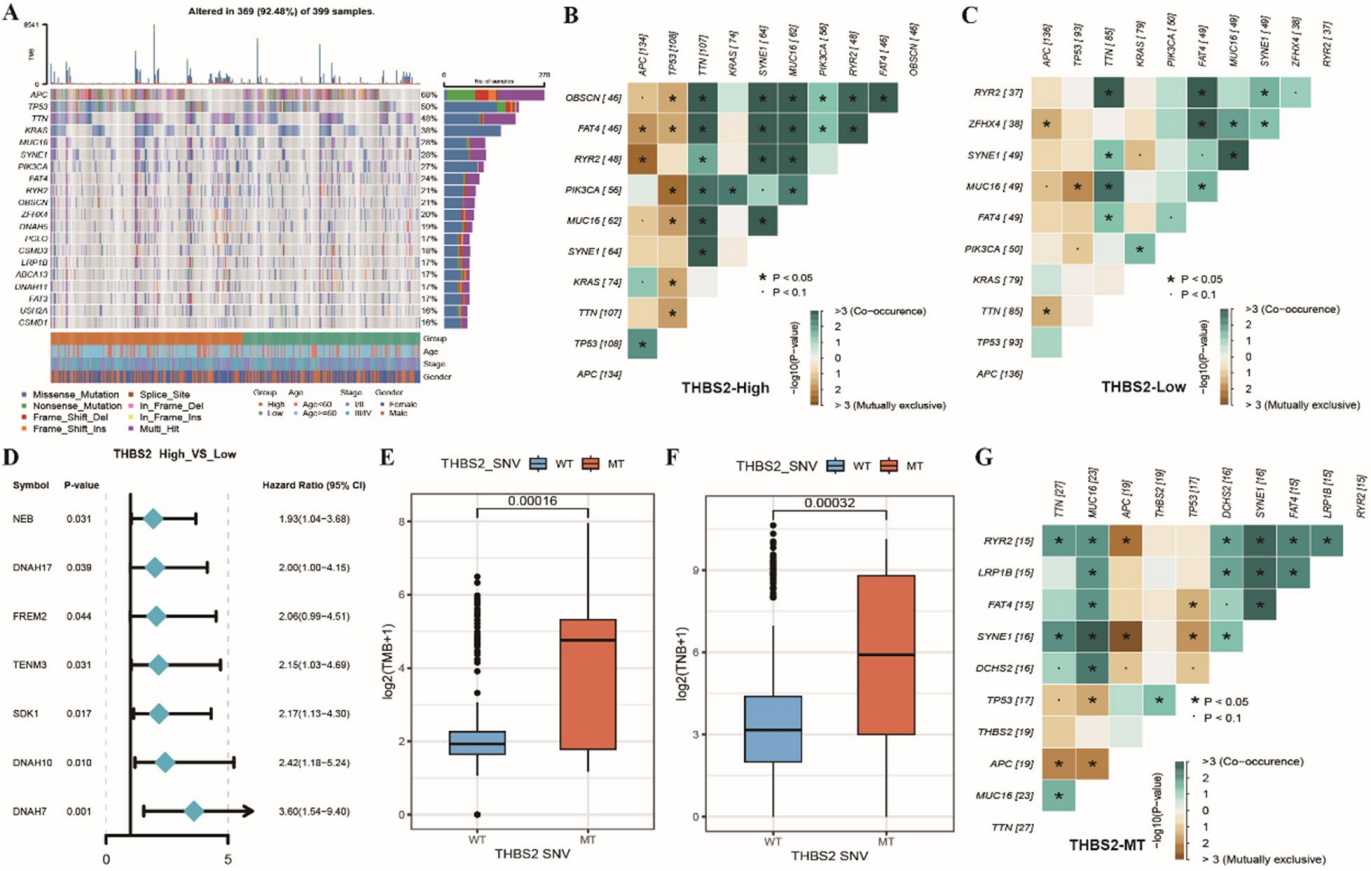
Genomic mutations associated with THBS2 in COAD. **A** Waterfall plot demonstrating the SNV frequency of top 20 genes in TCGA-COAD cohort; **B** Heat map demonstrating the co-linearity and mutual exclusivity of top 10 mutated genes in the high-THBS2-expression group; **C** Heat map demonstrating the co-linearity and mutual exclusivity of top 10 mutated genes in the low-THBS2-expression group; **D** Forest plot demonstrating differences in the mutation frequency of top 100 genes between the high- and low-THBS2-expression groups, with *p-*values representing significant differences; **E**, **F** Box plots demonstrating differences in TMB and TNB between the mutant-THBS2 (MT) and wildtype-THBS2 (WT) groups; **G** Heat map demonstrating the co-linearity and mutual exclusivity of top 10 mutated genes in the MT group

Gene mutations can promote, cause or orchestrate the malignant progression of tumours, and the study of mutations at the genomic level is crucial for developing tumour-targeting drugs and novel therapies for treating cancer. A waterfall plot integrating clinical information (age, stage and sex) and the top 20 mutated genes in the two THBS2 groups was plotted. The results showed that the SNV frequency of APC, TP53 and TTN was remarkably high in colon cancer (Fig. 8A). Subsequently, heat maps demonstrating the co-linearity and mutual exclusivity of the top 10 mutated genes in the two groups were plotted. The results revealed that co-linearity and mutual exclusivity were higher in the high-THBS2-expression group than in the low-THBS2-expression group (Fig. 8B, C). Furthermore, forest plots of the top 100 mutated genes with significant differences between the two groups were plotted to demonstrate their *p*-values and upper and lower confidence intervals (Fig. 8D). All samples in TCGA-COAD cohort were divided into two groups, mutant-THBS2 (MT) and wildtype-THBS2 (WT), based on the SNV status of the THBS2 gene. Both TMB and TNB were found to be significantly higher in the MT group than in the WT group (Fig. 8E, F). The mutation frequency of the top 10 genes was highly co-linear and mutually exclusive in the MT group (Fig. 8G).

### THBS2 affects lactate metabolism in CT26 cells through HIF1

**Fig. 9:**
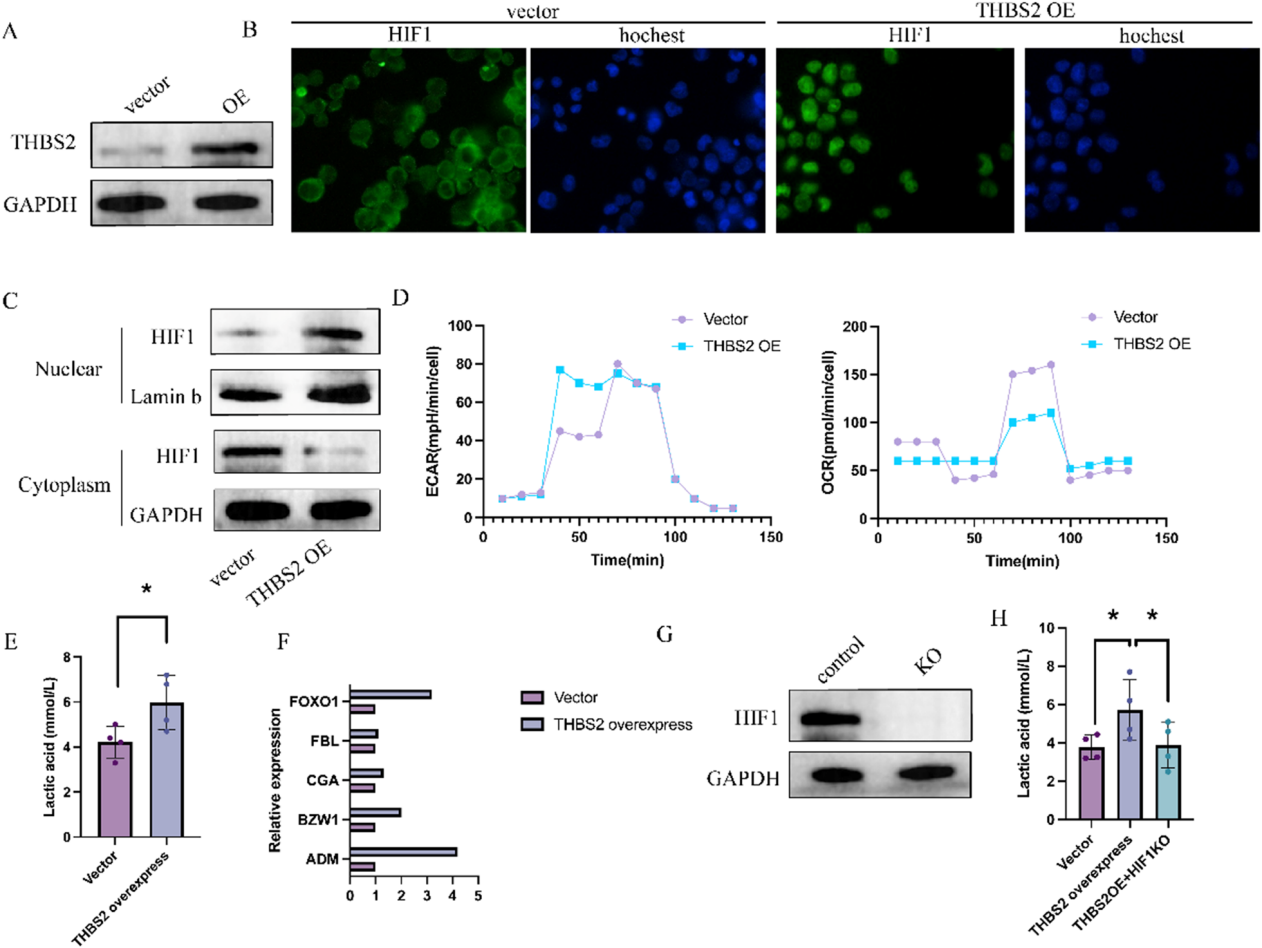
THBS2 influences lactate metabolism in CT26 cells through HIF1. **A** Representative images from three independent western blotting experiments showing the overexpression of THBS2 in CT26 cells. **B** Immunofluorescence staining of HIF1 (green) and nuclei (blue) in CT26 cells overexpressing THBS2. Representative images from three independent experiments are shown. **C** Detection of HIF1 expression in the nucleus and cytoplasm of CT26 cells overexpressing THBS2. **D** Measurement of extracellular acidification rate (ECAR) and oxygen consumption rate (OCR) in CT26 cells overexpressing THBS2 on a Seahorse analyser. **E** Assessment of lactate metabolism in CT26 cells overexpressing THBS2 (n = 3; *** indicates *p* < 0.001 compared with the vector group). **F** Detection of downstream transcriptional genes of HIF1 via PCR (n = 3; *** indicates *p* < 0.001 compared with the vector group). **G** Representative images of western blotting showing the knockdown of HIF1 in THBS2-overexpressing CT26 cells. Representative images from three independent experiments are shown. **H** Evaluation of lactate metabolism after THBS2 overexpression and HIF1 knockdown in CT26 cells (n = 3; ns indicates no significant difference compared with the vector group)

To investigate the relationship between THBS2 and HIF1, THBS2 was overexpressed in CT26 cells (Fig. 9A). Immunofluorescence analysis and western blotting revealed increased nuclear translocation of HIF1 in THBS2-overexpressing cells (Fig. 9B, C). As shown in Fig. 9D, THBS2 overexpression led to an increase in ECAR and a decrease in OCR in CT26 cells, indicating a shift toward anaerobic metabolism instead of oxidative phosphorylation for energy production. Consistently, THBS2 overexpression increased lactate production in CT26 cells (Fig. 9E). These results suggest that THBS2-overexpressing CT26 cells tend to derive energy through anaerobic glycolysis and produce lactate.

HIF1 regulates several genes involved in oxygen supply and metabolic adaptation. When stabilised and translocated to the nucleus, HIF1 binds to specific DNA sequences and initiates the transcription of target genes [29–31]. As shown in Fig. 9F, THBS2 overexpression enhanced the transcription of downstream genes regulated by HIF1. To rescue THBS2 overexpression-induced anaerobic glycolysis, HIF1 was knocked down in THBS2-overexpressing CT26 cells (Fig. 9G). The results revealed that HIF1 knockdown reversed the THBS2 overexpression-induced increase in lactate levels (Fig. 9H). Altogether, these results suggest that THBS2 enhances anaerobic glycolysis in tumour cells by modulating the nuclear translocation of HIF1.

### THBS2 influences M2 polarisation of macrophages through GPR132

We have previously reported that upregulation of THBS2 in CT26 cells leads to a shift toward anaerobic glycolysis and lactate production. Lactate can interact with GPR132 to activate downstream signalling pathways and impact macrophage function and polarisation [32]. Particularly, in the context of inflammation and the tumour microenvironment, high levels of lactate can promote the polarisation of macrophages toward the M2 phenotype. Therefore, in this study, we investigated whether upregulation of THBS2 in tumour cells exerts similar effects on macrophages.

**Fig. 10:**
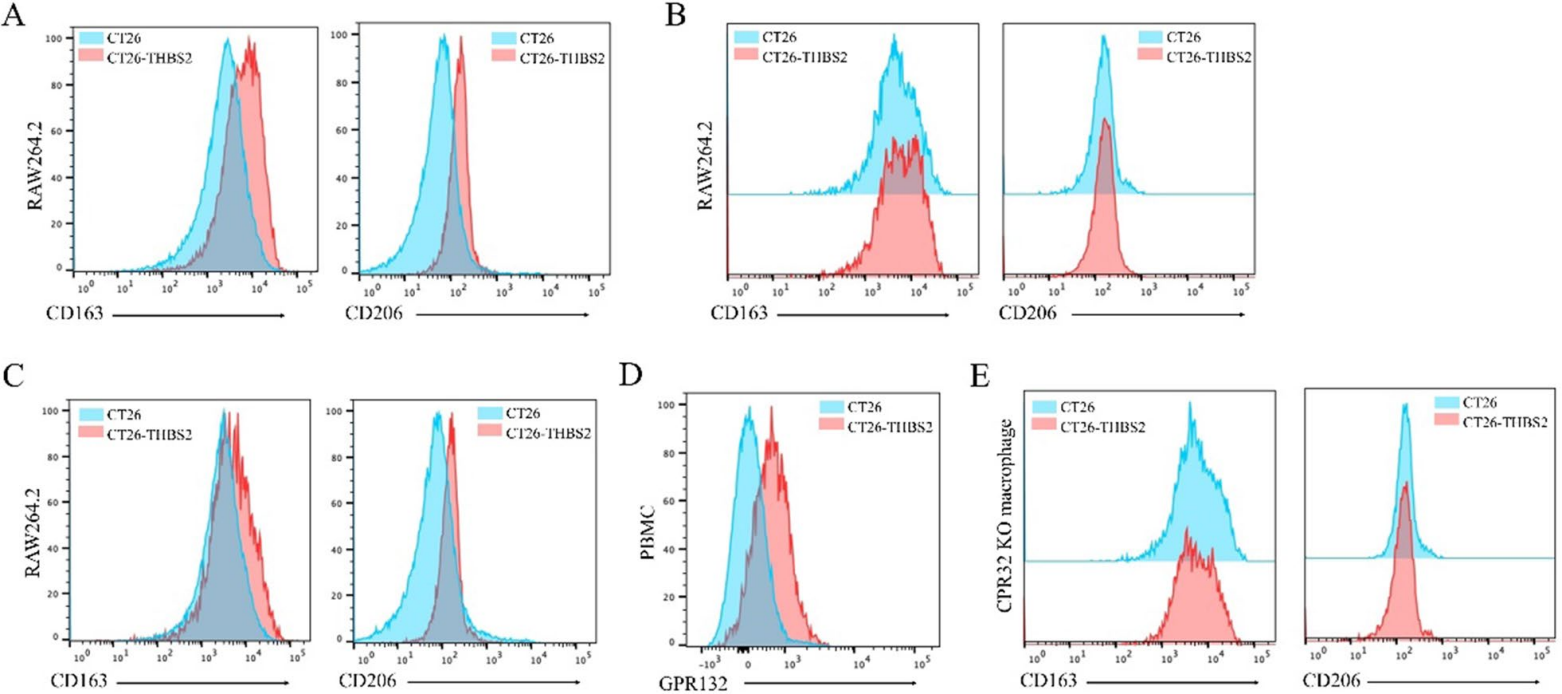
THBS2 influences M2 polarisation of macrophages through GPR132. **A** Collection of conditioned medium from THBS2-overexpressing CT26 cells and its addition to RAW264.7 cells. Flow cytometric analysis of CD163 and CD206 expression in cells after 24 h; **B** Centrifugation of conditioned medium from CT26 cells to isolate high-molecular-weight proteins and the addition of the medium to RAW264.7 cells. Flow cytometric analysis of CD163 and CD206 expression in cells after 24 h; **C** Addition of lactate to RAW264.7 cell culture medium. Flow cytometric analysis of CD163 and CD206 expression in cells after 24 h. **D** Flow cytometric analysis of GPR132 expression in peripheral blood mononuclear cells from GPR132-knockout (KO) mice; **E** Collection of conditioned medium from THBS2-overexpressing CT26 cells and its addition to macrophages from GPR132-KO mice. Flow cytometric analysis of CD163 and CD206 expression in cells after 24 h

RAW264.7 macrophages were co-cultured with the conditioned medium from THBS2-overexpressing CT26 cells. Flow cytometry revealed that the co-cultured cells exhibited an M2-like phenotype (Fig. 10A). The conditioned medium from CT26 cells was centrifuged to isolate high-molecular-weight proteins. These proteins failed to replicate the M2 polarisation-promoting effect (Fig. 10B). However, the addition of lactate to the culture medium of RAW264.7 cells facilitated their differentiation (Fig. 10C), suggesting that lactate is crucial for macrophage polarisation. To verify this hypothesis, GPR132-knockout (KO) mouse models were established, and peripheral blood monocytes were phenotypically characterised using flow cytometry (Fig. 10D). Macrophages isolated from GPR132-KO mice were co-cultured, demonstrating that GPR132-deficient macrophages did not respond to lactate induction (Fig. 10E). Altogether, these results indicate that THBS2 influences M2 polarisation of macrophages by interacting with GPR132.

### THBS2 overexpression in tumour cells mediates M2 polarisation of macrophages, resulting in the suppression of proliferation and cytotoxicity of CD8 + T cells

**Fig. 11:**
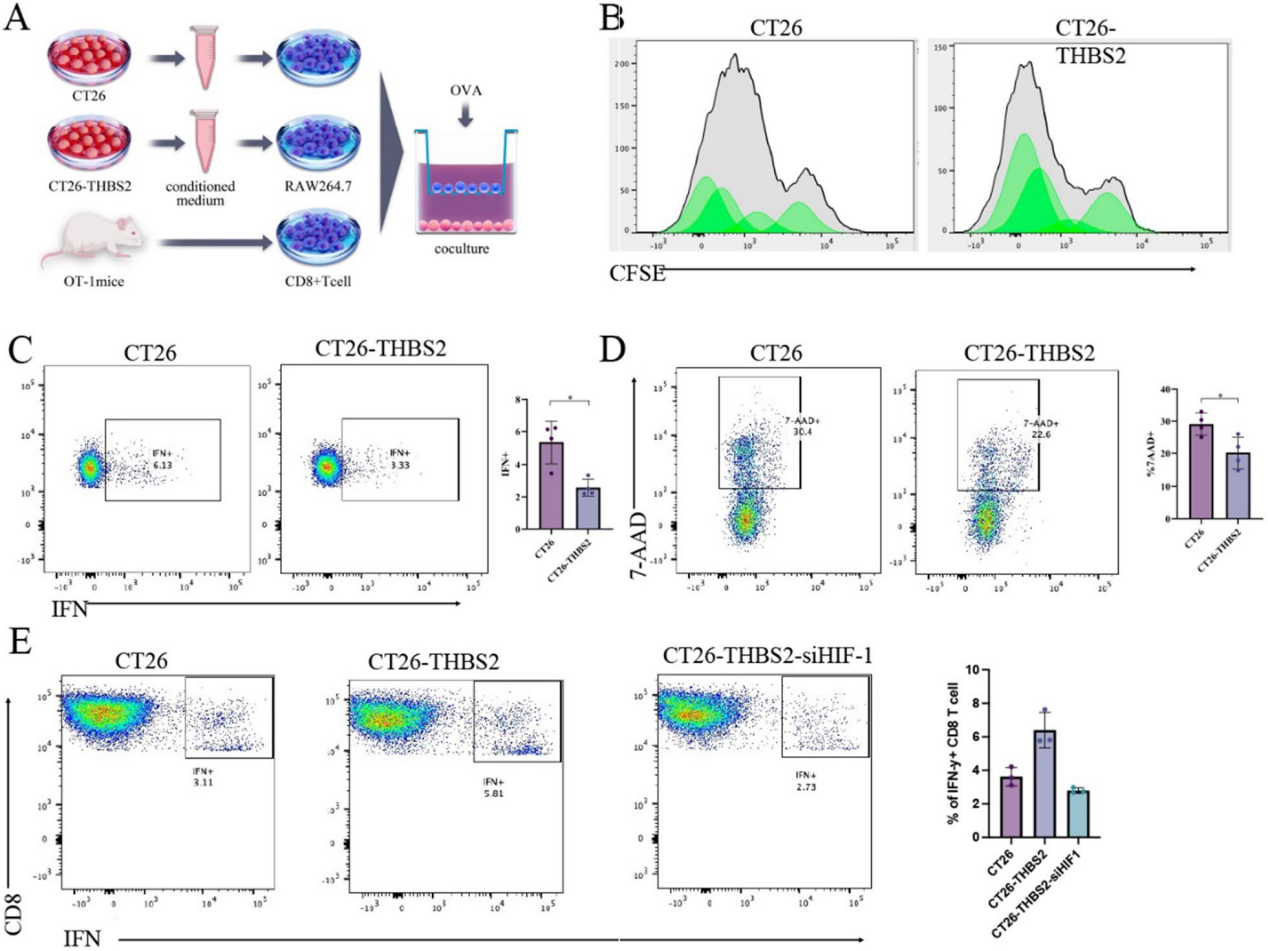
M2 macrophages suppress the proliferation and cytotoxicity of CD8 + T cells. **A** Schematic representation of co-culture of OT-1 cells with macrophages; **B** Flow cytometric analysis of CFSE in CD8 + T cells; **C** Flow cytometric analysis of IFN in CD8 + T cells; **D** Cytotoxic effects of CD8 + T cells on CT26 cells overexpressing OVA. Flow cytometric analysis of 7-AAD in CT26 cells. **E** IFN-γ released from OT-1 CD8 T cells

To assess the role of THBS2 overexpression in the tumour microenvironment, its impact on T-cell immune responses and anti-tumour immunity was examined in OT-1 mice. The transgenic T-cell receptor (TCR) in OT-1 mice recognises and responds to ovalbumin (OVA) antigen with high specificity. As shown in Fig. 11A, CD8 + T cells from OT-1 mice were co-cultured with M2-polarised macrophages influenced by THBS2-overexpressing CT26 cells from previous experiments. CFSE assay revealed that the proliferation of CD8 + T cells was significantly suppressed in response to OVA antigen when the cells were co-cultured with THBS2-CT26-influenced (M2-polarised) macrophages (Fig. 11B). Additionally, the release of IFN-γ was reduced (Fig. 11C). The cytotoxicity assay against CT26-OVA cells (CT26 cells overexpressing OVA antigen) demonstrated a significant killing effect of CD8 + T cells from OT-1 mice, whereas cytotoxicity was significantly diminished when influenced by M2-polarised macrophages (Fig. 11D). Furthermore, HIF1 was knocked down in CT26-THBS2 cells (CT26-THBS2-siHIF1 cells), and conditioned media derived from CT26, CT26-THBS2 and CT26-THBS2-siHIF1 cells were used to induce RAW264.7 polarisation. The macrophages were subsequently co-cultured with CD8 + T cells from OT-1 mice in the presence of OVA antigen. As shown in Fig. 11E, the release of IFN-γ decreased after THBS2 was overexpressed in CT26 cells and increased after HIF-1 was knocked down in THBS2-overexpressing CT26 cells. Altogether, these results indicated that M2 polarisation induced by THBS2-overexpressing CT26 cells significantly impaired the proliferation and cytotoxic potential of CD8 + T cells.

### THBS2 influences the infiltration levels, proliferation and cytotoxicity of T cells in the tumour microenvironment

**Fig. 12:**
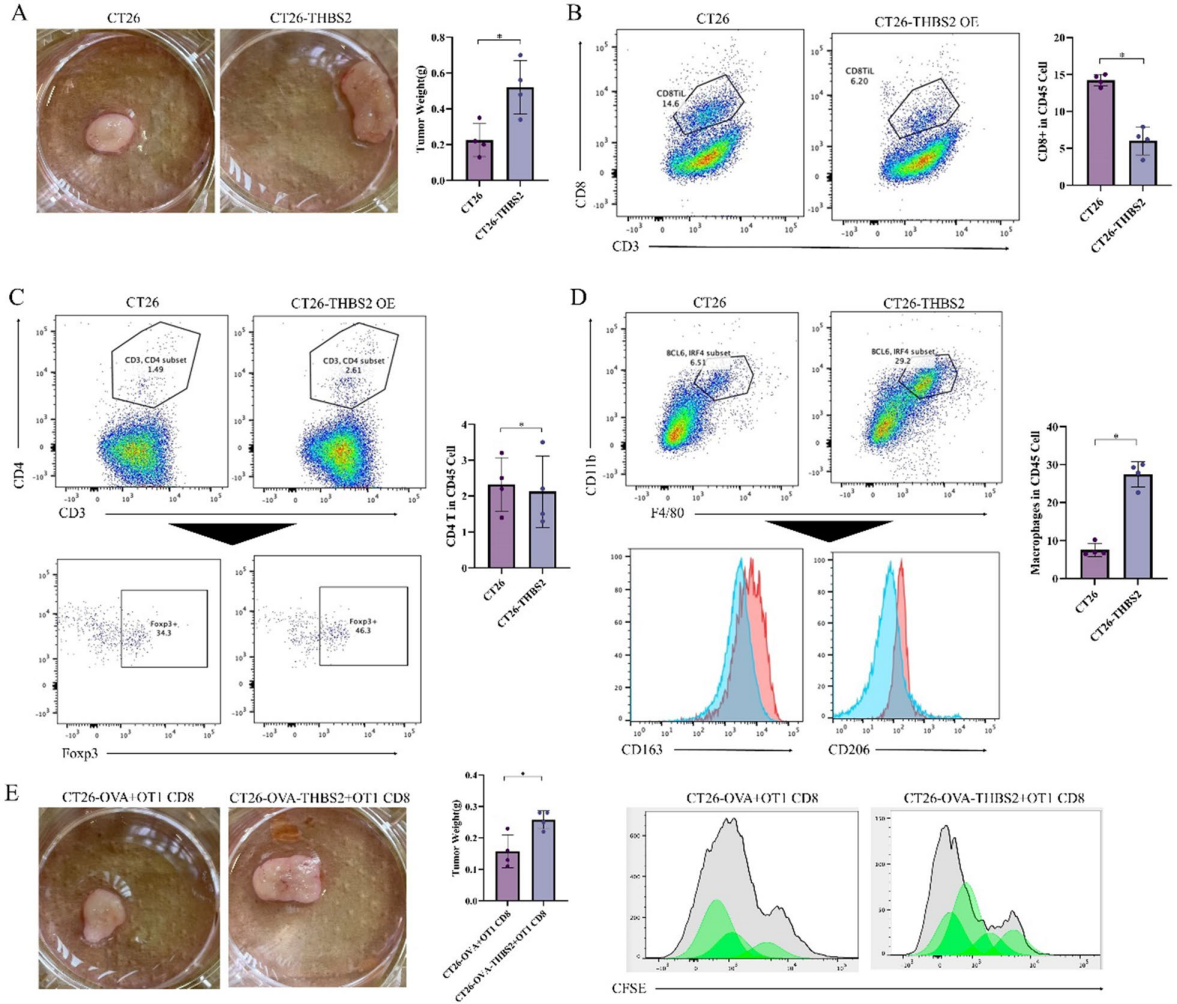
THBS2 influences the infiltration levels, proliferation and cytotoxicity of T cells in the tumour microenvironment. **A** Establishment of tumour-bearing Balb/c mouse models using CT26 and CT26-THBS2 cells. Tumour size was measured after 30 days; **B** Flow cytometric analysis of CD3 + /CD8 + cell abundance in tumour tissues of different groups; **C** Flow cytometric analysis of CD3 + /CD4 + and Foxp3 + cell abundance in tumour tissues of different groups; **D** Flow cytometric analysis of F4/80 + /CD11b + , CD163 + and CD206 + cell abundance in tumour tissues of different groups; **E** Implantation of CT26-OVA and CT26-OVA-THBS2 cells into Balb/c mice, followed by intravenous injection of CD8 + T cells from OT-1 mice for treatment. Tumour size was measured on day 0; F: Flow cytometric analysis of CFSE in tumour tissues

To examine the effects of THBS2 on the tumour microenvironment, CT26 cells and THBS2-overexpressing CT26 cells (CT26-THBS2) were injected into Balb/c mice. As shown in Fig. 12A, CT26-THBS2 tumour-bearing mice had significantly high tumour volume. In addition, these mice had a high abundance of CD3 + /CD8 + cells, indicating high infiltration levels of cytotoxic T lymphocytes (Fig. 12B). The increased abundance of CD3 + /CD4 + and Foxp3 + cells suggested increased infiltration levels of helper T lymphocytes and regulatory T cells, respectively (Fig. 12C). Additionally, CT26-THBS2 tumour-bearing mice had high macrophage infiltration, with a higher proportion of M2-polarised macrophages (Fig. 12D). After CT26 cells overexpressing OVA antigen (CT26-OVA) and CT26 cells overexpressing both OVA antigen and THBS2 (CT26-OVA-THBS2) were implanted into Balb/c mice, the mice were intravenously injected with CD8 + T cells from OT-1 mice. Treatment with CD8 + T cells significantly reduced tumour volume, whereas the impact on THBS2-overexpressing tumour cells was minimal (Fig. 12E). CFSE assay revealed that the proliferation of CD8 + T cells was considerably reduced in the CT26-OVA-THBS2 group (Fig. 12F), indicating that THBS2 inhibited the proliferation of CD8 + T cells in vivo.

### Immunosuppressive phenotype induced by high expression of THBS2 depends on GPR132 expression

**Fig. 13:**
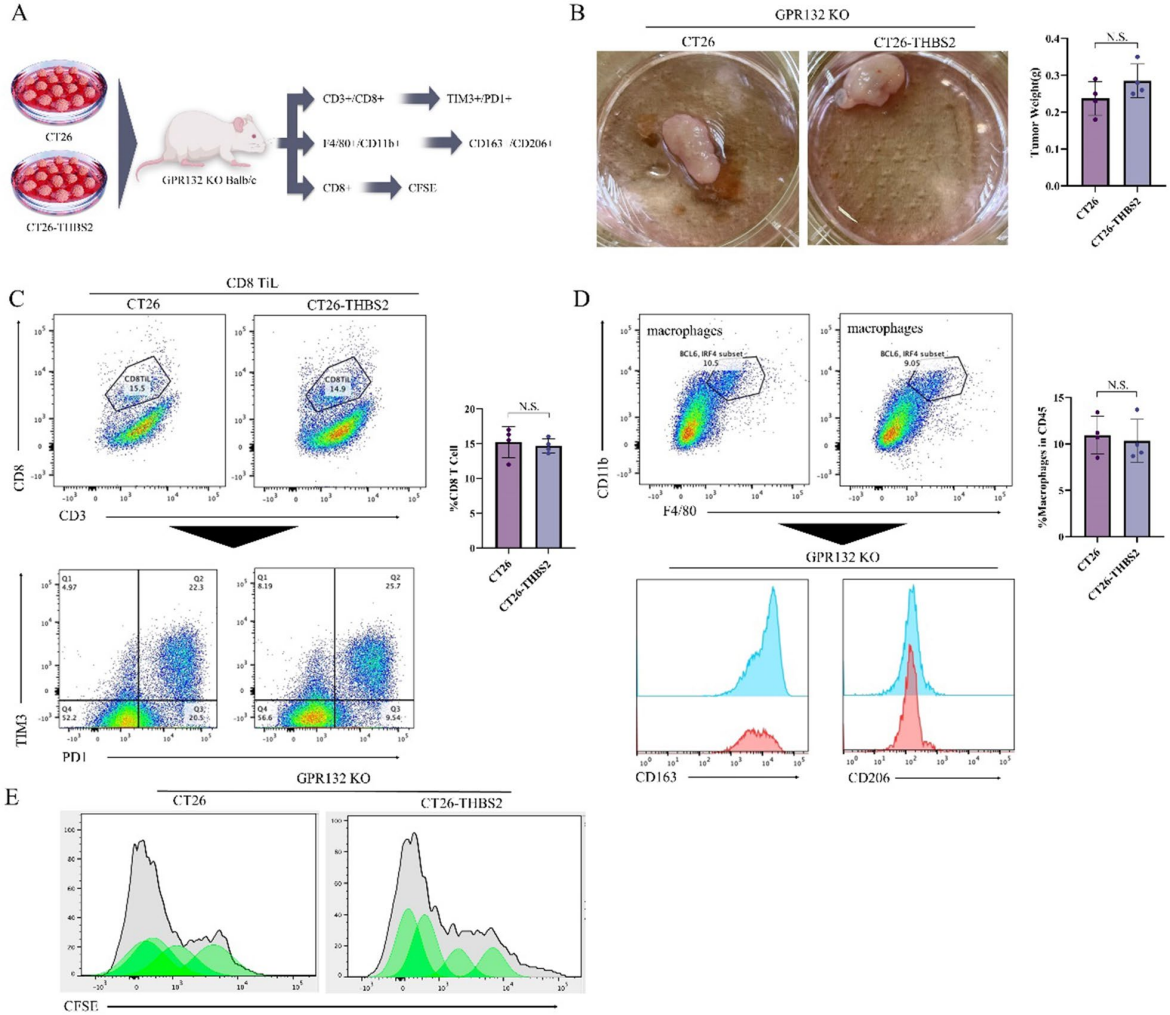
Immunosuppressive phenotype induced by high expression of THBS2 depends on GPR132 expression. **A** Establishment of GPR132-KO and tumour-bearing Balb/c mouse models; **B** Assessment of tumour size 30 days after the injection of CT26 and CT26-THBS2 cells in mice; **C** Flow cytometric analysis of CD3 + /CD8 + and TIM3 + /PD1 + cell abundance in tumour tissues of different groups. **D** Flow cytometric analysis of F4/80 + /CD11b + , CD163 + and CD206 + cell abundance in tumour tissues of different groups; **E** Flow cytometric analysis of CFSE in CD8 + T cells

To investigate the relationship between THBS2 and GPR132 in vivo, GPR132-KO Balb/c mouse models were established and inoculated with CT26 and CT26-THBS2 cells to induce tumour formation (Fig. 13A). The results showed that overexpression of THBS2 did not significantly alter tumour volume in GPR132-KO mice (Fig. 13B). In addition, the abundance of tumour-infiltrating CD8 + cells or TIM3 + /PD1 + CD8 + T cells was not substantially different across various groups of mice, indicating that the functional exhaustion of cytotoxic T lymphocytes in the tumour was not significantly affected (Fig. 13C). Similarly, no significant differences were observed in macrophage infiltration, M2 polarisation and CFSE dilution levels of CD8 + T cells across groups (Fig. 13D, E). These results suggested that GPR132 knockout blocked tumour progression and immunosuppression induced by THBS2-overexpressing CT26 cells.

## Discussion

With more than 18.1 million new cases and 9.6 million deaths reported annually, cancer has emerged as the second leading cause of death worldwide [33]. Genomic mutations and epigenetic variations enable tumour cells to acquire features such as metabolic abnormalities and immune escape [34, 35]. THBS2 acts as a platelet-responsive protein that mediates cell–cell and cell–extracellular matrix interactions. It can promote CRC cell migration and invasion through the Wnt/β-catenin signalling pathway [36]. In addition, it is upregulated in gastric cancer and is closely associated with immune cell infiltration [37]. THBS2 recombinant protein inhibits T-cell proliferation in vitro and promotes tumour growth and distant metastasis in lung adenocarcinoma in vivo [38]. Although the role of THBS2 in the abovementioned tumours has been frequently reported, no studies have investigated its role in pan-cancer. Pan-cancer analysis using public databases can be effective in understanding the molecular mechanisms and prognostic value of genes in tumours, thus improving and expanding clinical diagnostic and therapeutic strategies [39, 40]. In this study, we examined the expression, mutations and methylation levels of THBS2 and its relationship with immune cell infiltration in pan-cancer, demonstrating that THBS2 affects tumour prognosis through the aforementioned mechanisms. Furthermore, the expression, function and genomic mutations of THBS2 were comprehensively characterised in TCGA-COAD cohort. The results revealed that THBS2 expression was considerably associated with PFI, immune cell infiltration, immune regulation, cell death, cell migration, epithelial–mesenchymal transition, angiogenesis and genomic variations in COAD. In addition, THBS2 expression was associated with immune responses, indicating its potential as a target for immunotherapy in COAD.

Several in vitro and in vivo experiments were performed to investigate the mechanisms through which THBS2 contributes to the malignant progression of COAD. The results revealed that THBS2 modulated lactate metabolism in CT26 cells by regulating the HIF1 pathway. Overexpression of THBS2 led to upregulation of HIF1, consequently influencing intracellular lactate production and metabolism. Moreover, overexpressed THBS2 influenced the M2 polarisation of macrophages by interacting with GPR132, thereby affecting immune cell infiltration in the tumour microenvironment. Altogether, overexpression of THBS2 in CT26 cells resulted in HIF1 upregulation, subsequently altering cellular lactate metabolism. This metabolic alteration may be implicated in tumour growth and progression, as lactate metabolism has been associated with these processes.

The conditioned medium derived from THBS2-overexpressing CT26 cells promoted the differentiation of RAW264.7 cells into M2-like macrophages but failed to induce M2 polarisation in GPR132-deficient macrophages. These results suggest that GPR132 plays an essential role in mediating THBS2-induced M2 polarisation. Further analysis revealed that M2 macrophages suppressed the proliferation and cytotoxic capacity of CD8 + T cells. The co-culture of CD8 + T cells and M2 macrophages resulted in reduced proliferation of CD8 + T cells and downregulated IFN expression in these cells. Additionally, M2 macrophages inhibited the cytotoxic effects of CD8 + T cells on tumour cells. GPR132, also known as OxGR1 or G2A, functions as a seven-transmembrane G protein-coupled receptor capable of binding metabolic byproducts and lipid molecules, including lactate [32, 41]. Lactate interacts with GPR132 to activate downstream signalling pathways, influencing macrophage function and polarisation, particularly in inflammatory and tumour microenvironments [42, 43]. In this study, the interaction between THBS2 and GPR132 was assessed in an immunosuppressive tumour microenvironment. In GPR132-KO mice, overexpression of THBS2 neither altered tumour volume nor affected the abundance of tumour-infiltrating CD8 + T cells and exhausted CD8 + T cells. These results suggest that GPR132 is a critical mediator of THBS2-induced tumour progression and immune suppression.

In conclusion, this study reveals the pivotal role of THBS2 in the tumour microenvironment, highlighting its involvement in regulating lactate metabolism and M2 polarisation. Additionally, this study demonstrates that GPR132 is involved in mediating the impact of THBS2. These findings enhance the understanding of the functions of THBS2 and GPR132 in tumour immune regulation and provide potential targets for the development of immunotherapeutic strategies. However, further investigation is warranted to validate the therapeutic potential of THBS2 and GPR132 in cancer.

### Supplementary Information

**Additional file 1. **Functional enrichment analysis of down-regulated genes.

## Data Availability

The datasets used and/or analyzed during the current study are available with the corresponding author upon reasonable request.
