# Supplementary figures and images for: Systematic analysis of integrated bioinformatics to identify upregulated THBS2 expression in colorectal cancer cells inhibiting tumour immunity through the HIF1A/Lactic Acid/GPR132 pathway
Source: Cancer Cell Int. 2023 Oct 27;23:253. doi: 10.1186/s12935-023-03103-5 (PMC10604812; doi:10.1186/s12935-023-03103-5)

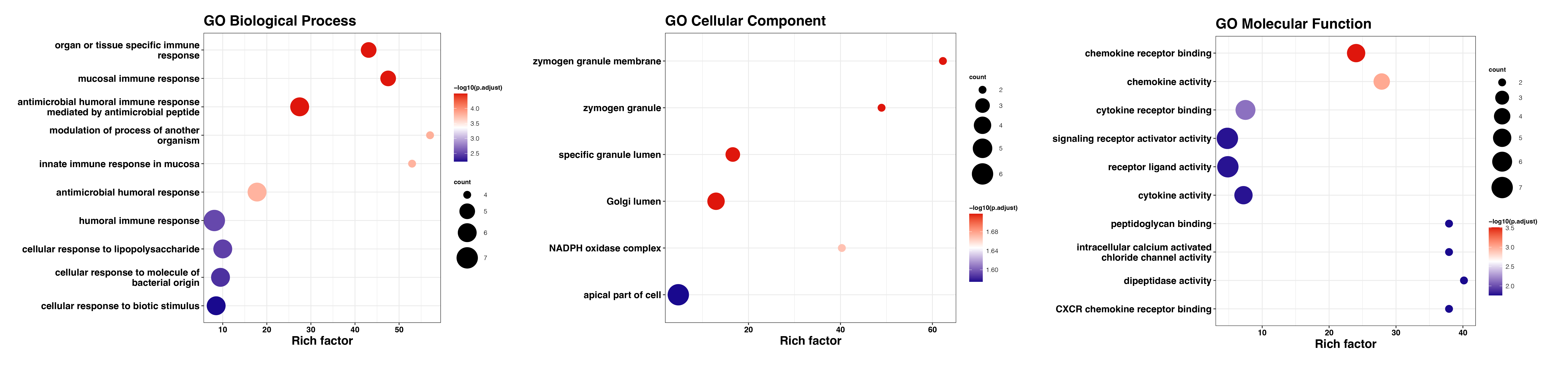

Supplement: Supplementary file 1 — Additional file 1. Functional enrichment analysis of down-regulated genes. [file 12935_2023_3103_MOESM1_ESM.jpg]
